# Cold-water immersion and other forms of cryotherapy: physiological changes potentially affecting recovery from high-intensity exercise

**DOI:** 10.1186/2046-7648-2-26

**Published:** 2013-09-01

**Authors:** Gillian E White, Greg D Wells

**Affiliations:** 1 Graduate Department of Exercise Sciences, The University of Toronto, Toronto, Ontario M5S 2W6, Canada; 2 Faculty of Kinesiology and Physical Education, The University of Toronto, Toronto, Ontario M5S 2W6, Canada; 3 Physiology and Experimental Medicine, The Hospital for Sick Children, Toronto, Ontario M5S 2W6, Canada

**Keywords:** Recovery, Performance, Cold therapy, Muscle damage, Injury, Mechanical stress, Metabolic stress

## Abstract

High-intensity exercise is associated with mechanical and/or metabolic stresses that lead to reduced performance capacity of skeletal muscle, soreness and inflammation. Cold-water immersion and other forms of cryotherapy are commonly used following a high-intensity bout of exercise to speed recovery. Cryotherapy in its various forms has been used in this capacity for a number of years; however, the mechanisms underlying its recovery effects post-exercise remain elusive. The fundamental change induced by cold therapy is a reduction in tissue temperature, which subsequently exerts local effects on blood flow, cell swelling and metabolism and neural conductance velocity. Systemically, cold therapy causes core temperature reduction and cardiovascular and endocrine changes. A major hindrance to defining guidelines for best practice for the use of the various forms of cryotherapy is an incongruity between mechanistic studies investigating these physiological changes induced by cold and applied studies investigating the functional effects of cold for recovery from high-intensity exercise. When possible, studies investigating the functional recovery effects of cold therapy for recovery from exercise should concomitantly measure intramuscular temperature and relevant temperature-dependent physiological changes induced by this type of recovery strategy. This review will discuss the acute physiological changes induced by various cryotherapy modalities that may affect recovery in the hours to days (<5 days) that follow high-intensity exercise.

## Review

### Introduction to cryotherapy

Cryotherapy includes whole body cryotherapy (dry air of −80°C to −110°C for 1–3 min), cold-water immersion (CWI), ice or cold gel pack application, ice massage or any other local or general application of cold for therapeutic purposes [1]. Although these types of treatments are commonly and ubiquitously used to speed recovery from stressful bouts of exercise, no standard guidelines have been established, and a target temperature for optimal therapeutic effects has yet to be identified [2,3]. This is largely owing to a lack of understanding regarding the mechanisms through which cryotherapy affects recovery from high intensity exercise [4]. Of the many forms of cryotherapy used to this end, CWI is the most popular in the literature and in practice [2]. Several studies have investigated [5-11] and reviewed [2,3,12-14] the effects of CWI for reducing soreness and speeding the recovery of force-generating capacity by skeletal muscles following stressful bouts of exercise. However, evidence regarding the efficacy of CWI, and cryotherapy in general, to speed recovery remains equivocal. Many reviews have concluded that the high heterogeneity in methodology regarding exercise insult, cold protocol and performance outcomes are responsible for the current lack of agreement in the literature [2,3,13,14]. This review will investigate physiological changes as potential mechanisms induced by cryotherapy modalities of all kinds used for the purpose of reducing tissue temperature to facilitate acute (1 h–5 days) recovery from high-intensity exercise. Although we recognize the importance of long-term effects of chronic use of an anti-inflammatory intervention, such as cryotherapy, the physiological changes induced by cryotherapy will not be discussed in this context.

### Introduction to exercise stress

Exercise that is novel, highly eccentric or of particular intensity or duration induces unaccustomed stress on the body. Depending on the specific nature of the exercise, the stress may be predominantly metabolic, mechanical or a mix of both [3,12,15]. The mechanisms implicated in exercise-induced muscle damage and inflammation have been well-reviewed [15-19]. In brief, exercise inducing primarily metabolic stress in active skeletal muscles, such as endurance or interval training, involves a high rate of aerobic energy transformation [20] and heat generation [21]. Both contribute to an increase in reactive oxygen species (ROS) generation. ROS are highly reactive and can denature proteins, nucleic acids and lipids, which destabilize muscle cell structures including the sarcolemma [22] and structures of the excitation–contraction coupling system [23]. Damage to the excitation–contraction coupling system alters contraction kinetics, thereby reducing force-generating capacity and athletic performance, while disruption of the sarcolemma makes the muscle fibre more permeable [17]. The sustained high transformation of energy to support repeated contractions and increased intramuscular pressure imposed by hyperaemia [24] can also impose mild hypoxic stress on the muscle fibre promoting the accumulation of metabolites [16]. The accumulation of metabolites within the cell caused by high metabolic rate increases the osmolality of the cell. Paired with increased permeability, the potential for cell swelling (oedema) is enhanced [25,26]. Oedema increases mechanical stress on cell structures, increases the route for O_2_ delivery, and compresses capillaries, impairing O_2_ delivery and waste removal, as well as causes soreness [27]. Systemically, this type of exercise also challenges the cardiovascular and neuromuscular systems. Ultimately, an increase in cytosolic calcium concentration within the muscle fibre leads to the activation of proteases [18,28,29] and signalling of inflammatory cells [28], as well as promotes oedema [29]. Coupled with the damage by ROS and muscle fibre swelling, an exercise-induced inflammatory response is initiated. Although inflammation is required for resolution of any muscle fibre damage resulting from the exercise insult, if excessive or unabated, the phagocytotic activity of neutrophils and macrophages contribute to secondary muscle damage [30]. Secondary muscle damage, damage incurred by the inflammatory response to exercise and not the exercise bout *per se*, compounds the soreness and reduction in force-generating capacity experienced in the hours and days following a high-intensity exercise bout. Cold may facilitate recovery from metabolically stressful exercise by reducing intramuscular temperature and metabolism [31] to ease hypoxic stress, limit ROS generation and subsequent damage [25,32], as well as by inducing vasoconstriction to limit oedema formation [4,31,33] and associated damage and soreness.

**Figure 1:**
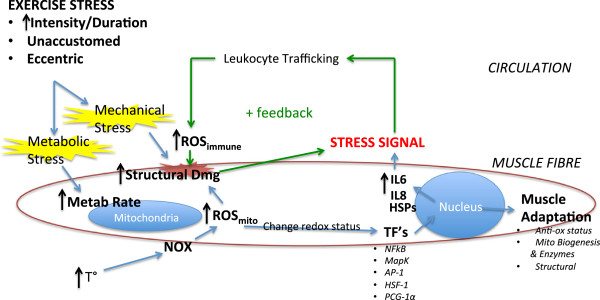
**Exercise**-**induced cell signalling.** High intensity or high duration exercise induces metabolic stress and increases reactive oxygen species (ROS) production at the mitochondria of skeletal muscle, which contributes to lipid peroxidation and structural cell damage, as well as alters the redox status of the cell. Several transcription factors (TFs), such as nuclear factor kappa B (NFκB), Map Kinase (MapK), activator protein-1 (AP-1), heat shock factor protein-1 (HSF-1), and peroxisome proliferator-activated receptor-γ coactivator (PCG)-1α, are redox sensitive; thus, their function may be altered by the change in redox status. Some of these TFs are involved in muscle adaptation pathways, while others are involved in the production and secretion of cell signalling molecules such as interleukin-6 (IL-6) and interleukin-8 (IL-8). These cytokines are involved in the trafficking of leukocytes, which are attracted to the cell to clear away damaged tissue, but they may also contribute to ROS production at the muscle cell, contributing to structural damage and propagating the positive feedback pattern of the inflammatory response. Similarly, mechanical stress, such as that induced by high force contraction or highly eccentric exercise, may directly cause structural damage, initiating a similar positive feedback mechanism, but attracting leukocytes, which produce ROS and compound structural damage incurred. Lastly, high temperatures induced by exercise may increase the production of ROS from NADPH oxidase (NOX), contributing to the structural damage, change in redox status, nuclear signalling and positive feedback signalling associated with the other forms of exercise stress.

Mechanical stress imposed on active skeletal muscle during exercise that includes high force contractions, such as plyometrics or resistance training can cause direct physical disruption of the sarcolemma, sarcomeres, excitation–contraction coupling system, and connective tissue associated with the muscle [34]. High intramuscular pressures, sustained release of calcium from the sarcoplasmic reticulum, and strain induced by force-producing cross-bridges contribute to mechanical stress during high-force skeletal muscle contractions [17,35]. Typically, eccentric exercise evokes a comparatively greater degree of mechanical stress and subsequent muscle damage compared with concentrically or isometrically biased exercise [36]. This is due to the combination of cross-bridges producing force while lengthening, greater force per muscle fibre, and the large degree of force contribution by passive tissues [16]. A loss of structural integrity of the sarcolemma and contractile system is directly induced by the strain experienced during contractions in response to mechanical stress. Sarcolemmal disruption enhances cell permeability and swelling, while disruption to the excitation–contraction coupling system impairs force-producing capability, and both contribute to soreness and reduced function. Loss of calcium homeostasis within the cell initiates protease activity and subsequent damage [17]. Damage to cells, oedema and cytokines released from the muscle exposed to mechanical stress initiate an inflammatory response, increasing the potential for secondary damage and resultant impairment of function. The application of cold cannot reduce the initial damage incurred by the muscle during this type of exercise. However, reduced neural conductance velocity (NCV) limits spasm and pain sensation [37], while local vasoconstriction reduces swelling [4,25,31], inflammatory signalling and the potential for secondary damage to the muscle fibres [27]. Figure 1 shows a summary of the damaging events initiated by exercise stress.

Using cryotherapy immediately following exercise is thought to reduce undesirable reductions in muscle performance and increases in muscle soreness that are commonly experienced in the hours/days following exercise. Cold is accepted as a temporary analgesic [1,14,27,37,38]. As an anti-inflammatory, it may reduce the stimulus inciting the activation of pathways that cause secondary damage [32,39]. Reducing the ultimate damage incurred by the muscle reduces the magnitude of repair necessary to achieve pre-exercise structural and functional integrity, thereby shortening recovery time. Although ROS production and the inflammatory response contribute to secondary damage of injured/stressed muscle fibre cells [32,39], they also play a crucial role in cell signalling for the remodelling and adaptation of skeletal [40-42]. Thus, a paradox between the use of cryotherapy for acute reduction in inflammation to facilitate recovery and the potential negative effects caused by blunting the stress response may exist. This review will focus only on the acute physiological responses to cryotherapy and their potentially therapeutic effects.

### Physiological changes as potential mechanisms for therapeutic effects

Many of the physiological changes suspected to be integral to the therapeutic effects of cryotherapy are thought to be temperature-dependent. Thus, methodological factors affecting the tissue temperature achieved by the various methods of cryotherapy is of particular importance. The literature has shown magnitude of tissue temperature change is positively correlated with cryotherapy methods that undergo a phase change [43], present a larger thermal gradient [44], are of longer duration [9], are applied to a larger surface area [45], or employ a greater mass of medium [45], while increased subcutaneous adiposity is negatively correlated with reduction in intramuscular temperature [46,47]. Despite the differences in the rate of temperature change, the pattern of skin and intramuscular temperature change between modalities seems to be consistent. Skin temperature drops rapidly in the first 1–3 min and reaches minimum temperature around 8–9 min of cooling [45]. Superficial intramuscular temperature cools in a linear pattern at a rate faster than deeper muscle tissues [48], with the magnitude of muscle temperature change being dependent on the thermal gradient between the muscle and cryotherapy medium [48]. Although both superficial and deep intramuscular tissues reach a minimum temperature in the post-cooling period, deeper tissues will reach a nadir in temperature later in the post-cooling period as heat from deep tissues is lost to colder superficial tissues [48-50]. This has important implications concerning the rationale for using intermittent cooling strategies vs. continuous cooling strategies. Potential for damage when using cryotherapy is related to skin and superficial tissue temperature achieved, as these tissues reach significantly lower temperatures than muscle (i.e. skin temperature values of 6.5 ± 3.4°C vs. muscle temperature values of 27.8 ± 3.5°C 1 cm below subcutaneous level) [43] and contain a greater number of vulnerable tissues (i.e. nerves). Using intermittent cooling may allow muscle to reach colder temperatures while limiting the potential for damage since muscle continues to cool during the post-application period, while skin rapidly rewarms. Curiously, no reports of freezing or non-freezing cold injury have been reported; however, individuals with a history of these afflictions such as frostbite, chilblains, etc. should be cautious when considering the use of cryotherapy [51].

**Figure 2:**
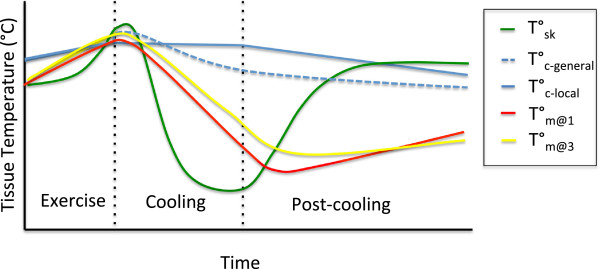
**Relative pattern of temperature change in different tissue layers during exercise**, **cooling and post**-**cooling period.** Data are averaged from studies measuring changes in tissue temperatures using various forms of cryotherapy [4,9,43,45-48,54,55]. Skin temperature (green, *T*_sk_) increases during exercise, decreases exponentially through cryotherapy, reaching nadir earliest, and increases exponentially through post-cooling period. Core temperature (blue dashed, *T*_c-general_) changes induced by cryotherapy applied to large mass increases during exercise, and decreases during cryotherapy (rate dependent on thermal gradient and peripheral blood flow). Core temperature cools slower than other tissues and does not begin to return to baseline until 1 h post-cooling. Core temperature (blue solid, *T*_c-local_) changes induced by cryotherapy applied to a small mass is minor during cryotherapy and modest throughout post-cooling period as blood cooled at periphery is returned to core. Superficial intramuscular temperature (red, *T*_m@1_) increases during exercise, declines linearly during cryotherapy, and increases linearly to baseline within 1 h. Deeper intramuscular temperature (yellow, *T*_m@3_) increases during exercise decreases linearly during cryotherapy at a lower rate than *T*_m@1_, continues to cool through the post-cooling phase as heat is transferred to warmer superficial tissues, returning to baseline later than 1 h.

Cryotherapy, especially CWI, is commonly used to attenuate hyperthermia by reducing core temperature following exercise in hot environments. Methodological variables affecting peripheral tissue temperature also affect core temperature change. However, the pattern and magnitude of change differs due to the increased reliance of core temperature change on convective cooling as opposed to conductive cooling [52]. With local cryotherapy that induces cutaneous vasoconstriction (skin temperature = 21°C–12°C) [52], core temperature decreases linearly to a modest extent due to vasoconstriction at the cold periphery limiting the exposure of blood to cold tissues. With a greater body mass exposed to cold, conductive cooling causes a substantial reduction in core temperature while convective heat loss is minimal during the application period [52]. Following cooling in both local and general cryotherapy, rewarming of skin causes subcutaneous vasodilation, which enhances the flow of cooled blood from the tissues cooled by cryotherapy back to the core, resulting in an enhanced reduction in core temperature [4]. During more modest cryotherapy with large mass exposed to cold (i.e. lower body immersion in 21°C–27°C water), sustained blood flow to skin allows for convective cooling during the application period and further cooling in the post-cooling period. However, the rate of core cooling is slightly lower (0.8°C min^−1^ vs. 1.0°C min^−1^) as conductive heat loss due to the lower thermal gradient is reduced [52]. It is also important to note that rate of change in core temperature observed in hyperthermic individuals is greater (i.e. following high-intensity exercise or activity in hot environments) as a greater thermal gradient is present [53]. Change in temperature of tissues stressed by exercise is the fundamental physiological change induced by cooling and a determining factor in the subsequent physiological changes affecting recovery from exercise. Figure 2 shows the generalized relative patterns of change in skin, superficial muscle, deep muscle and core temperature induced by local cooling with various modalities (values are averaged between studies to show relative patterns of change).

#### Tissue metabolism

Following exercise, stressed muscle fibres may have an increased energy demand as they restore ion gradients, repair structural damage and replace energy stores [56]. Thus, reducing muscle energy demand by cooling may reduce the metabolic stress experienced by a muscle fibre by minimizing the disparity between O_2_ supply and demand. Respiratory chain function of mitochondria contributes significantly to ROS production of a skeletal muscle cell [57]. Thus, reducing the rate of mitochondrial energy production by depressing intramuscular temperature can be expected to limit ROS-mediated damage incurred by stressful exercise. Studies have shown reduced perfusion and oxygen usage by muscles when cooled at rest [44,58] and following exercise [31] suggesting a reduced aerobic metabolic rate; however, no human studies have investigated the effects of post-exercise muscle cooling on ROS. Animal studies using muscle strain models have shown significant reductions in ROS-mediated damage and an overall reduction in the inflammatory response when cold was applied compared with no cold application [39]. However, muscle temperatures were not recorded, and a muscle strain model may not accurately reflect the magnitude of muscle fibre damage and stress resulting from exercise.

Some studies have shown enhanced metabolite clearance and reduced metabolic waste accumulation, including inorganic phosphate and hydrogen ions [25]. When Yanagisawa et al. [25] used ^31^P MR spectroscopy to investigate the effects of 15 min of CWI at 5°C following strenuous exercise, they observed muscle pH values to be higher (pH = 7.2) than those for muscle that was not cooled (pH = 7.0) 60 min post-exercise. Muscle acidosis is a contributing factor in the development of peripheral fatigue and impairment of force-generating capacity of muscle [59] and may be relieved by cold therapy post-exercise, possibly contributing to short-term recovery enhancement. However, this finding should be viewed with discretion as any beneficial effect of cold-induced pH restoration could be concealed by the reduced functional capacity of a muscle that is cold [60]. As rates of reaction are reduced with lower temperature, it is interesting to note that muscle glycogen resynthesis post-exercise does not seem to be affected by CWI (8°C for 10 min) following exercise (total glycogen synthesis at 4 h was 83 ± 43 and 79 ± 58 mmol kg^−1^ dw for control and CWI, respectively) [61]. This is a surprising finding given that glycogen resynthesis rates are expected to be negatively affected by reduced metabolic rate of muscle fibres. Subjects were given carbohydrate supplementation at 30 min, 1, 2, and 3 h; thus, it is possible that glycogen synthesis was compensated for when muscle temperature and blood flow had been restored post-cooling. Alternatively, as the carbohydrate supplementation was relatively low (0.6 g/kg body weight), it may be that the glycogen resynthesis was not optimized in the non-cooling condition. More research is needed in this area as this is an important aspect of recovery for many types of athlete.

#### Blood flow

Cooling causes reflexive vasoconstriction, due to increased affinity of alpha-adrenergic receptors for norepinephrine in the vascular walls [62] and is thought to contribute to the anti-inflammatory influence of cold during recovery. Although reducing blood flow to stressed/fatigued muscles to enhance recovery seems counterintuitive, muscle fibres with sarcolemmal disruption or with increased osmolality have increased risk of oedema. Additionally, the hyperaemic effect of exercise increases intramuscular pressures [24], which may independently impair O_2_ delivery. Thus, reducing blood flow is thought to decrease muscle fibre oedema and subsequent pain, functional impairment and potential for secondary damage caused by inflammation. However, colder skin temperatures (i.e. <10°C) may cause a cold-induced vasodilatory reflex [63], such that blood is shunted from muscle to subcutaneous tissue with minimal change in whole limb blood flow [64]. Studies using strain gauge plethysmography or other forms of whole limb volume assessments of muscle haemodynamic changes should be interpreted with caution. Gregson et al. [4] used laser Doppler (cutaneous blood velocity) and duplex ultrasound (femoral artery blood flow) during 10 min (2 × 5 min) of immersion in 8°C or 22°C water. Whole limb blood flow was reduced by approximately 40% in both conditions; however, cutaneous blood flow was unchanged from baseline during cooling in the 8°C condition, while it was significantly reduced in the 22°C condition. The authors concluded that muscle blood flow was reduced more in the colder condition due to redistribution of flow to the cutaneous tissues. While both conditions showed changes to blood flow, the contribution of hydrostatic forces of water immersion in this particular study are unknown as no thermoneutral immersion condition was used as a control condition. Studies have shown a tendency for intramuscular blood flow to remain depressed [48] or continue to decrease during the post-cooling period [31,64], likely because intramuscular temperature remains below baseline for hours following cooling. A study investigating cold-induced blood flow changes following exercise observed similar results to studies measuring changes with cooling at rest. However, the magnitude of blood flow reduction was more modest with cooling following exercise (20% reduction from control following exercise vs. 40% at rest) [31,64]. Therefore, altered blood flow is an important mechanism through which CWI may enhance recovery following high-intensity exercise; however, the effects of reducing blood flow to reduce oedema and the implications this has for O_2_ delivery post-exercise have not been elucidated. Further, the long-term effect of chronic cryotherapy use should be considered as vascular remodelling following an exercise stimulus that has been shown to be impaired [65].

#### Fluid shunting and oedema

Oedema caused by increased permeability of the sarcolemma causes pain, functional decrements and impaired O_2_ delivery. Cold reduces blood flow to compromised muscle fibres, which decreases the potential for swelling of these cells. Additionally, compressive forces commonly combined with cold (i.e. hydrostatic forces of water, wraps used with ice bags, etc.) structurally limit swelling and fluid accumulation, while facilitating removal of wastes and increasing central blood volume. Exercise-induced muscle oedema seems to be biphasic, such that an initial increase in intracellular volume occurs acutely (0–2 h) post-exercise due to osmotic shifts (metabolite accumulation and initial damage), while a sub-acute increase (24–96 h) may be attributed to increased permeability resulting from inflammatory mediated secondary membrane damage [31]. Using T2-weighted magnetic resonance imaging (MRI) images, Yanagisawa et al. [33] observed that sub-acute muscle oedema (T2 value difference of 7.3% ± 5.6% between the control and CWI condition was observed at 96 h) and activity of creatine kinase, an indirect marker of muscle damage, were attenuated by immersion in 5°C water for 15 min. In a subsequent study, using flow-sensitive MRI, a 4%–6% increase in signal intensity was observed in the control limb dorsiflexors compared with no significant increase in the limb treated with an ice pack [66]. However, the relative contributions of vasoconstriction and hydrostatic forces are not clear because a thermoneutral condition was not used. Humoral and vascular indices affected by 10°C, 20°C or 32°C water immersion indicate that the effects of immersion alone may be humoral, whereas changes induced by cold are likely related to altered affinity of vascular adrenergic receptors [67]. Cold applications of excessive duration (>30 min) or low temperature (<10°C) may increase oedema, as they have been shown to result in cold damage to cells [68].

#### Neuromuscular effects

Soreness caused by stressful exercise can impair performance while microtrauma to muscle fibres can promote muscle spasm. NCV has a direct linear correlation with temperature [69]; thus, cooling reduces NCV of both sensory and motor neurons, reducing the sensation of pain and reflexive spasm, respectively. Herrera et al. [37] found CWI to be the most effective cooling modality to reduce motor and sensory NCV in the 30-min post-cooling period. Interestingly, sensory and motor neural conductances seem to be affected differently. Sensory neurons are influenced by more modest changes in temperature, possibly due to their superficial anatomic location [70]. Thus, cooling may have an analgesic effect before motor neurons are significantly affected, allowing for mobility with minimal pain sensation. Cooling skeletal muscle impairs contraction kinetics in non-fatigued muscle [60,69,71], specifically rates of tension development, indicating that the rate of excitation–contraction coupling is impaired. It is therefore important to consider the effects of reduced muscle temperature when interpreting performance outcomes or using cold between consecutive exercise bouts, as the muscle may still be at sub-physiological temperature.

#### Cardiovascular effects

Both partial (up to waist) [38,72] and head-out (up to neck) [9,10,38] CWI can alter neural activity of the heart, as well as restore central blood volume and enhance cardiac preload. Both of these cardiovascular indices have been suggested to improve recovery from metabolically stressful exercise. Several studies have shown improved cardiac efficiency, as indicated by lower heart rates, increased cardiac output and/or increased stroke volume [7,73-75]. Park et al. [75] observed a roughly 50% increase in cardiac output in subjects in water vs. subjects at rest in air, which the authors attributed to increased stroke volume and cardiac preload. A greater increase in stroke volume was observed in colder water, which may be a result of cold-induced vasoconstriction, while increased stroke volume in thermoneutral water may be due solely to the compressive hydrostatic forces of water. Studies investigating the effects of CWI on blood volume distribution and subsequent exercise performance showed faster heart rate recovery and increased performance in a second bout of exercise (1 h later) compared with active recovery [10].

CWI may restore vagal tone and normalize parasympathetic modulation of heart rate following intense exercise [11,72-74]. Heart rate recovery and heart rate variability indices seem to be improved by immersion of subjects in thermoneutral (34°C) or CWI (15°C) temperatures compared to no immersion [73]. Water immersion in cold temperatures appears to be effective in enhancing cardiovascular measures of recovery through increased venous return and cardiac output and through a more efficient return to baseline neural activity of the heart. Cardiovascular changes are likely only induced by cryotherapy modalities that expose a large proportion of body mass to cooling medium. Although cardiovascular function altered by CWI may be theoretically beneficial, especially between bouts of metabolically stressful exercise, few studies [10] have investigated the significance of these changes with respect to the recovery of performance *per se*.

#### Endocrine

As both exercise and cold can be recognized as stress by the body, changes in circulating hormone concentrations may be elicited. These hormones modulate blood flow, fluid balance, heart rate and breathing frequency, among other physiological parameters that may be relevant during recovery from high-intensity exercise. Few studies have specifically looked at the changes in circulating hormone levels when CWI is used for recovery compared with passive or other types of recovery. Srámek et al. [67] measured changes in circulating renin, aldosterone, cortisol, norepinephrine, epinephrine and dopamine induced by immersion in 14°C, 20°C or 32°C water for 1 h and observed a trend towards decreased cortisol following both the 14°C and 20°C conditions and increased concentration of norepinephrine and dopamine following only the 14°C condition. Increased diuresis was observed in all conditions, increasing with lower temperatures of immersion. Although this does not describe the modulation of circulating hormones following exercise, it does suggest that water immersion is capable of altering some key endocrine factors; however, the physiological significance of these changes is unknown. Further, systemic endocrine changes are likely limited to forms of cryotherapy that expose a large mass of body to a cold medium as shown in Table 1.

**Table 1:** Summary of tissue temperature changes induced by various forms of cryotherapy and subsequent changes in potential physiological mechanisms

	**Method**	***T***_**sk**_	***T***_**m**_		***T***_**c**_		**Oxygen use**	**Blood flow (%Δ from baseline)**		**HR**	**Oedema**	**Applications/conclusions**	**References**
			**Superficial**	**Deep**	**0 h**	**Nadir**		**Skin**	**IM**				
Ice pack	0.3–1.8 kg crushed ice	5°C–7.5°C	26.58°C^a^–27.9°C	28.21°C^a^–31.82°C							↓46% fair signal intensity	Limited performance or function data may reduce oedema post-eccentric exercise	[43,45,46,54,55,66]
		Δ −25.0°C–30°C	Δ −7.0°C–9.7°C	Δ −4.46°C–8.38°C									
		(20–30 min)	(5–10 min post-cool)	(10–15 min post-cool)									
Cold pack application (no phase change)	0°C–10°C for 20–30 min	7.9°C–22.5°C	15°C–24.8°C						↓20%–40%		↓0.822% ∆ in MRI T2 values	No performance data	[44,48,64]
		Δ −12°C–23°C	Δ −9.7°C–18°C										
		(20–30 min)	(20 min)										
CWI partial immersion	0°C–12°C for 3–15 min	11°C–15.1°C	20°C^a^–30.4°C	33.3°C^a^–34.0°C	37.12°C	37.11°C^a^		↓24**%** (20 min post-cool)	↓30%–40% (30 min post-cool)	↑5–10 bpm (1st minute of cooling)	↓2.5% ∆ in MRI T2 value (48 h)	Post-eccentric—equivocal	[4,25,33,38]
		Δ −14.5°C–20°C	Δ −13.6°C–15.0°C	Δ −2.0°C–4.3°C	Δ −0.11°C	Δ −0.23°C						Post-high-intensity interval—somewhat beneficial	
		(2–10 min)	(5–30 min post-cool)	(5–60 min post-cool)	(0 h)	(30 min post-cool)						Lacking performance studies that measure temperature or mechanisms	
CWI partial immersion	12°C–21°C for 3–60 min	17°C–26°C	32.2°C^a^	34.3°C^a^–34.5°C	36.3°C–37.6°C	36.2°C^a^–37.3°C						Post-eccentric—equivocal	[6,9,38,52,53,72]
		Δ −7°C–17.2°C	Δ not recorded	Δ −1.6°C–1.8°C	Δ −0.01–6°C	Δ −0.1–0.4°C						Post-high-intensity interval—somewhat beneficial	
		(3–60 min)	(30 min post-cool)	(30–60 min post-cool)	(0 h)	(30 min post-cool)	↓2.5 tissue oxygenation index (12 min)		↓20 (15 min)			Lacking performance studies that measure temperature or mechanisms	
CWI head-out immersion	14–15°C for 14–20 min			32.3°C^a^	34.7°C–37.7°C	36.0°C^a^–36.8°C			↓76% from post-exercise	↓12 pm (post-cool vs. active recovery)	↓0.70025 mid-thigh girth	Maintains or improves performance in bouts of exhaustive exercise (i.e. cycling time trial or sprint) that are within hours, when used as a between bout recovery	[9-11,38,67]
				Δ −5.9°C	Δ −0.9°C–1.3°C	Δ −1.8°C–2.0°C						Not studied following eccentric or high-intensity interval exercise	
				(35 min post-cool)	(0 h)	(15–30 min post-cool)							
Whole body cryotherapy	−110°C for 3–4 min	17.0		34.5°C^a^	37.8°C–37.9°C	37.2°C^a^–37.5°C						No performance differences reported	[55,76]
		Δ −13.2°C		Δ −1.6°C	Δ −0.3°C–0.07°C	Δ −0.25°C–0.3°C							
		(0–1 min)		(60 min post-cool)	(0 h)	(3–8 min post-cool)						Reductions in inflammatory markers have been shown	

Methodologies within each form of cryotherapy are variables. Ranges for duration of application, temperature and/or mass of cooling medium are given where applicable. ‘*T*_sk_’ denotes skin temperature, ‘*T*_**m**_**’** denotes muscle temperature, ‘*T*_c_’ denotes core temperature and ‘IM’ denotes intramuscular. For all temperature changes, the range is given, followed by change from baseline (denoted by delta ‘Δ’), and time at which these measurements were recorded (shown in brackets). ^a^Values were recorded in the post-cooling period.

## Conclusions

Similar events, including increased cytosolic calcium, sarcolemmal permeability, muscle fibre oedema, disruption to cell structures and resultant soreness and loss of force-generating capacity result from both metabolically and mechanically stressful exercises; however, the aetiology and extent of muscle fibre damage, temporal sequence of events and magnitude of inflammatory response differ. Table 1 shows a summary of tissue temperature changes induced by different types of cryotherapy and the subsequent changes in potential physiological mechanisms. The majority of applied studies using cryotherapy for recovery from exercise cite its effectiveness as a by-product of its ability to blunt inflammation through reducing local metabolism and inducing vasoconstriction. Although metabolic rate and blood flow seem to be reliably affected by cold, studies have yet to investigate a dose-dependence of cold on inflammation, the downstream and, arguably, more important outcome of applying cold. Such a variety of methodological approaches to studying cold presents a challenge to drawing reasonable conclusions from a mechanistic point of view. Further, very few studies measure tissue temperature change while investigating either physiological or muscle functional responses to cryotherapy following exercise. Lack of temperature data in addition to the wide variety of exercise stress protocols used to study cryotherapy for recovery has resulted in general disagreement with respect to what types of exercise might benefit from cryotherapy and which method of cryotherapy may be the most appropriate. As different types of exercise induce different stress responses, the recovery necessary to attain a pre-exercise state is different. This must be considered in future studies as cold is not likely to affect recovery from all types of exercise uniformly and thus may not be appropriate for all types of exercise. Given its widespread use, this is of particular importance. It is possible that since stressful exercise, both predominantly metabolically or mechanically stressful, induces inflammation which contributes to secondary damage, the ability of cold to blunt the initial stress signal stimulating inflammation is common to both types of exercise stress. Although changes in local metabolism, blood flow and oedema and systemic changes in cardiovascular, neuromuscular and endocrine function are altered by cryotherapy following stressful exercise, few studies concomitantly study these physiological responses speculated to be mechanistic in the recovery effect of cryotherapy and inflammatory and/or functional outcomes. Thus, although these physiological changes are induced by lowering tissue temperature and may have a role in facilitating recovery from some types of exercise, studies investigating the mechanisms concomitant with functional outcomes are needed to substantiate whether cryotherapy has an effect greater than simply a placebo or subjective improvement in recovery.

## Abbreviations

AP-1: Activator protein-1; CWI: Cold-water immersion; HSF-1: Heat shock factor protein-1; IL-6: Interleukin-6; IL-8: Interleukin-8; MapK: Map Kinase; NCV: Neural conductance velocity; NFκB: Nuclear factor kappa B; PGC-1α: Peroxisome proliferator-activated receptor-γ coactivator (PCG)-1α; ROS: Reactive oxygen species; TF: Transcription factor.

## Competing interests

The authors declared that they have no competing interests.

## Authors' contributions

GEW reviewed the literature, organized and wrote the article and conceptualized and compiled figures. GDW critically revised the article, consulted on general direction and material to be covered and approved the final copy of the article.

## Authors' information

Greg D. Wells, Ph.D is an Assistant Professor at the Faculty of Kinesiology and Physical Education and in the Department of Anesthesia at the University of Toronto where he directs the Human Physiology Research Unit. He is also an Associate Scientist in Physiology and Experimental Medicine at the Hospital for Sick Children. As a researcher, Dr. Wells is working in a field of investigation called ‘exercise medicine’ where he uses specific exercise protocols to test the limitations of the human body in various diseases mostly related to muscle and lung conditions.

Gillian E. White is a Master's of Science candidate at the Faculty of Kinesiology and Physical Education in the Human Physiology Research Unit. She is working in the field of exercise inflammation and recovery from high-intensity exercise with a specific interest in the effects of cryotherapy on recovery from high-intensity exercise.
